# Quantification of Bacterial Colonization in Dental Hard Tissues Using Optimized Molecular Biological Methods

**DOI:** 10.3389/fgene.2020.599137

**Published:** 2020-12-18

**Authors:** Torsten Sterzenbach, Anne Pioch, Martin Dannemann, Christian Hannig, Marie-Theres Weber

**Affiliations:** ^1^Clinic of Operative and Pediatric Dentistry, Medical Faculty Carl Gustav Carus, Technische Universität Dresden, Dresden, Germany; ^2^Institute of Lightweight Engineering and Polymer Technology (ILK), Technische Universität Dresden, Dresden, Germany

**Keywords:** root canals, dentistry, hydroxyapatite, DNA-isolation, qPCR, bacterial colonization, endodontology, irrigation

## Abstract

Bacterial infections of root canals and the surrounding dental hard tissue are still a challenge due to biofilm formation as well as the complex root canal anatomy. However, current methods for analyzing biofilm formation, bacterial colonization of root canals and dental hard tissue [e.g., scanning electron microscopy, confocal laser scanning microscopy (CLSM) or determination of colony forming units (CFU)] are time-consuming and only offer a selective qualitative or semi-quantitative analysis. The aim of the present study is the establishment of optimized molecular biological methods for DNA-isolation and quantification of bacterial colonization via quantitative PCR (qPCR) from dental hard tissue. Root canals of human premolars were colonized with *Enterococcus faecalis*. For isolation of DNA, teeth were then grinded with a cryo mill. Since the hard tissues dentin and especially enamel belong to the hardest materials in the human organism, the isolation of bacterial DNA from root dentin is very challenging. Therefore, treatment steps for the isolation of DNA from grinded teeth were systematically analyzed to allow improved recovery of bacterial DNA from dental hard tissues. Starting with the disintegration of the peptidoglycan-layer of bacterial cells, different lysozyme solutions were tested for efficacy. Furthermore, incubation times and concentrations of chelating agents such as EDTA were optimized. These solutions are crucial for the disintegration of teeth and hence improve the accessibility of bacterial DNA. The final step was the determination of prior bacterial colonization of each root canal as determined by qPCR and comparing the results to alternative methods such as CFU. As a result of this study, optimized procedures for bacterial DNA-isolation from teeth were established, which result in an increased recovery rate of bacterial DNA. This method allows a non-selective and straightforward procedure to quantify bacterial colonization from dental hard tissue. It can be easily adapted for other study types such as microbiome studies and for comparable tissues like bones.

## Introduction

Endodontic treatments are a frequent therapy in the daily dental practice. Since the number of retained teeth has increased around the globe, also the prevalence of endodontic diseases has grown especially within the elderly population (Persoon and Özok, 2017). During an endodontic treatment, the main goal is to relieve the patient from pain and remove the bacterially infected dentinal tissue (Plotino et al., 2016). Along with the mechanical preparation of the contaminated roots, different disinfective agents, and irrigation protocols are used to decontaminate the infected root canals (Plotino et al., 2016). However, persistent microbial infections in teeth regarding the root canals still cause dental endodontic therapies to fail and lead to the retreatment or even the loss of affected teeth (Lin et al., 1992; Endo et al., 2013; Tabassum and Khan, 2016). Therefore, different analytical methods have been implemented to test the effect of the existing disinfective irrigants on contaminated root canal systems and monitor microbial colonization *in vitro*. A very common method to detect possible residual microorganisms within root canals in *in vitro*-studies is the collection of contaminated debris as well as biofilm with paper points directly out of the root canal and the analysis via determination of colony-forming units (CFU) (Görduysus et al., 2012; Tan et al., 2015; Tzanetakis et al., 2015). Furthermore, microscopic methods like fluorescent microscopy, transmission electron microscopy (TEM), scanning electron microscopy (SEM), or cultivation are commonly used to evaluate bacterial colonization and biofilm formation (Herzog et al., 2017; Kirsch et al., 2017, 2019; Mohmmed et al., 2017; Ruiz-Linares et al., 2017; Tsesis et al., 2018). However, these methods are time-consuming and labor-intensive. Furthermore, they only offer selective qualitative or semi-quantitative analyses. Microscopic studies generally only visualize parts of the specimens. Likewise, the determination of CFU from dental hard tissue is challenging since it is difficult to completely collect microorganisms and biofilm from the root canal walls with paper points during *in vitro*-studies. Therefore, the described methods generally do not allow complete recovery of bacteria from the root canal system. Furthermore many species of the oral microbiome are difficult to cultivate or are yet uncultivable (Deo and Deshmukh, 2019).

Such caveats can be omitted by molecular biological methods during *in vitro*-studies. Hereby, bacterial DNA from cultivated samples is extracted. DNA content arising from targets of interest can then be amplified by quantitative PCR (qPCR) using specific oligonucleotides for priming (Kralik and Ricchi, 2017). Furthermore, such DNA should be suitable for next generation sequencing. In addition, these kinds of studies can be easily performed in high-throughput settings.

However, the root canal system consists of a complex anatomy with dentinal tubules, isthmi and complex apical structures. Due to the difficult canal anatomy it is impossible to shape and clean the root canal system completely (Cunningham and Martin, 1982; Shuping et al., 2000; Ferreira et al., 2004). Therefore, chemical agents in combination with ultra/-sonic devices serve as irrigating solutions to clean the untouched areas (Svec and Harrison, 1977; Gulabivala et al., 2010). However, it is difficult to transport the irrigant to these specific areas, especially in the apical part of the canal (Gu et al., 2009) and it is even more difficult to reach these bacteria by shaping files. This also makes bacteria or bacterial DNA hiding in the complex root anatomy hardly accessible for analytical methods. Therefore, the knowledge of an effective disinfection protocol is necessary and *in vitro*-studies which determine the cleansing effect are crucial. The benefit of *in vitro*-studies is the possibility to perform a targeted contamination of root canal systems and then evaluate, e.g., new irrigation protocols of disinfective agents or sonic/ultrasonic devices.

Therefore, reliable methods for robust DNA isolation of microbial DNA from dental hard tissue would be of great use for molecular biological quantification of bacterial loads after the targeted contamination and performed cleaning protocols.

However, enamel and dentin, the main dental hard tissues, are primarily composed of hydroxyapatite (≈90 and ≈75%, respectively), the mineral form of calcium apatite (Hannig and Hannig, 2010; Shellis et al., 2012). The large percentage of hydroxyapatite makes teeth the hardest material in the human or animal body (Maté Sánchez, de Val et al., 2016). This limits the accessibility of these materials for DNA purification procedures. Similar problems also arise with other materials containing high concentrations of hydroxyapatite like bones (Rohland and Hofreiter, 2007; Kattimani et al., 2016). Therefore, it is challenging to isolate high-quality and high amounts of DNA from these materials. Several methods exist to disrupt materials containing hydroxyapatite (teeth and bones). Mostly samples were disrupted by cryogenic milling (Alves et al., 2009; Özok et al., 2012; Antunes et al., 2015; Siqueira et al., 2016; Keskin et al., 2017; Qian et al., 2019). Hydroxyapatite can then be dissolved by several methods (e.g., strong mineral or weaker organic acids, chelating agents) (Yoshioka et al., 2002; Choube et al., 2018). The most common and gentle one is decalcification by chelating agents like ethylenediaminetetraacetic acid (EDTA) (Castania et al., 2015). EDTA binds calcium ions from the surface and thereby gently dissolves hydroxyapatite and hence increases accessibility of bacteria trapped within complex dental structures. DNA is then purified via column-based methods or isolation with organic solvents and precipitation (Ali et al., 2017). However, isolation procedures were generally not optimized. While it is not essential for qualitative studies to isolate bacterial DNA to maximum quantity, this is an essential trait for quantitative studies. Especially absolute quantification of bacterial colonization by quantitative PCR requires an almost complete recovery of DNA. In addition, microbiome studies are biased if DNA from less accessible sites is underrepresented (Pollock et al., 2018).

*E. faecalis* is the most commonly found bacterial species in recurrent root canal infections (Portenier et al., 2003). This is at least partly due to its capability to invade deep into dentinal tubulus (Kirsch et al., 2017, 2019). A major factor for this ability is the expression of adhesins mediating adherence to collagen which is the main organic component of dentin (Love, 2001). Furthermore, the expression of the adhesin Ace in conjunction with the presence of collagen promotes increased resistance to IKI, NaOCl and Ca(OH)_2_ (Kayaoglu et al., 2008). Therefore, we decided to use *E. faecalis* in this study as a model organism.

In this study, we optimized and validated DNA isolation procedures of microbial DNA from dental hard tissue and present a streamlined protocol for DNA isolation.

## Materials and Methods

### Chemicals, Teeth, and Bacterial Strains

*E. faecalis* ATCC 29212 was purchased from the DSMZ (German Collection of Microorganisms and Cell Cultures). Human premolars were obtained from Enretec GmbH (management facility for dental waste). Ethylenediaminetetraacetic acid (EDTA), sodium chloride (NaCl), thymol, and tris(hydroxymethyl)aminomethane (Tris)-HCl were purchased from Roth, lysozyme, and agarose were purchased from Sigma-Aldrich and Triton X-100 was purchased from Serva.

### Preparation of Human Premolars and Cultivation With *E. faecalis*

The procedure is essentially as previously described with some modifications (Kirsch et al., 2017). Suitable extracted human single-canal premolars were selected and stored in 0.1% thymol. Then the crowns of the teeth were separated from the roots and the roots were prepared with Pro Taper Gold F2 (Dentsply) under irrigation with sodium chloride (0.9%). Next, the roots were placed for 10 min in an ultrasonic bath with tryptic soy broth (TSB, Merck, Germany) to prepare the teeth for the bacterial incubation with *E. faecalis*. Then the roots were sterilized by autoclaving for 20 min in TSB and embedded with 3% agarose in 1.5 ml conical tubes (Eppendorf).

For inoculation of teeth root canals, *E. faecalis* was grown from a single colony for 16 h in TSB. Root canals of human premolars were then inoculated on two consecutive days with ≈1.5 × 10^8^ CFU/ml (≈10–20 μl per root canal depending on the size of the canals). Teeth were incubated aerobically at 37°C for 3 weeks unless indicated otherwise. Medium was exchanged every day. Teeth were grinded using a 6775 Freezer/Mill^®^ Cryogenic Grinder (SPEX^®^) with the following program: Precool 10 min, Run Time 1 min, Cool Time 1 min, Cycles 4, Impactor Rate 12. During the grinding procedure, teeth were constantly cooled with liquid nitrogen. The tooth powder was then transferred into 1.5 ml conical tubes.

### Microscopical Methods

Here the procedure is as described above except that two external grooves were prepared longitudinally on opposing sides along the roots, in order to be able to bisect the roots in halves. The grooves were filled with a lightbody a-silicon (Provil novo Light, Kulzer) to prevent bacteria to exit the root canal via dentinal tubules in the area of the grooves. One half of the roots was prepared for fluorescent microscopic analysis with DAPI (4′,6-diamidino-phenylindole)-staining to detect the bacterial DNA by binding to the AT-rich regions of the double stranded DNA (Kirsch et al., 2017). Upon binding to DNA, the DAPI molecule fluoresces intensely at λ = 461 nm. In order to perform the visualization technique with DAPI, the root halves needed to be decalcified with Osteosoft^®^ (Merck, Darmstadt, Germany) for 3 weeks. Every 3 days, the Osteosoft^®^-solution was changed until the root halves were sliceable with a scalpel. Then, the root halves were embedded in paraffin, followed by dehydration in an ascending series of ethanol. When fixed in Xylol (Carl Roth GmbH Co. KG, Karlsruhe, Germany), the root halves could be cut with a Microtome (Leica Biosystems Nussloch GmbH, Germany) into 2 μm slices. The thin slices were then mounted on top of a silanated object carrier. Afterward, the samples were rinsed with 0.9% sodium chloride. For staining, the samples were covered with DAPI solution (1.5 μl stock solution in 500 μl PBS (phosphate buffered saline) in the dark. After 15 min the DAPI solution was removed and the samples were rinsed several times with PBS before fluorescence microscopic analysis took place. The specimens were dried at room temperature and coated with Vectrashield mounting medium (Vectra laboratories, California, United States) and analyzed by epifluorescence microscopy (Axioplan, Zeiss, Oberkochen, Germany). The root canal samples with the dentinal tubules were analyzed at 1,000-fold magnification using the light filter for DAPI (BP 365, FT 395, LP 397). The area of ocular grid allowed visualization of the total length of the dentinal tubules (Kirsch et al., 2019).

The other half of the root canal was analyzed with scanning electron microscopy (Kirsch et al., 2019). Hereby, the root canal halves were fixed with glutaraldehyde for scanning electron microscopic investigation (SEM). Followed by dehydration in an ascending series of isopropanol and chemical drying through the iterative transfer into hexamethyldisilazane (HMDS), the samples were fixed on SEM stubs and were sputtered with gold-palladium. Scanning electron microscopy was carried out using a Philips ESEM XL 30 in Hi-Vacuum mode by detecting secondary electrons for imaging (Kirsch et al., 2019).

### Isolation of DNA

Unless indicated otherwise, 15 mg of tooth powder were dissolved in 200 μl of the, respectively, indicated solutions. For decalcification of the human premolars, the tooth powder was dissolved in the indicated solutions with varying EDTA concentrations and incubated under agitation at 37°C for the indicated amount of time. Then the powder was centrifuged at 8,000*g* for 30 s and the supernatant was transferred into a separate tube. The tooth powder was then dissolved in 180 μl of a solution containing 20 mg/ml lysozyme dissolved in 20 mM Tris-HCl pH 8.0, 1.2% Triton X-100 containing the indicated concentration of EDTA. The supernatants were treated separately and mixed with 180 μl of the indicated lysozyme-containing solution. Both the tooth powder and supernatants were then incubated under agitation for the indicated amount of time at 37°C. DNA was then isolated with the Relia Prep gDNA Tissue Miniprep System (Promega) according to the manufacturer’s protocol with the following modifications. Both the tooth powder and supernatant fractions were mixed with 1 volume of cell lysis buffer and 0.1 volumes of Proteinase K and incubated at 56°C for 2 h under agitation. The following steps were conducted according to the manufacturer’s protocol except that DNA was eluted with twice 50 μl H_2_O.

Genomic DNA of *E. faecalis* for generation of standard curves was isolated from an overnight culture using Relia Prep gDNA Tissue Miniprep System (Promega) following manufacturer’s instructions.

### Quantification of Bacterial Colonization

Quantitative real-time PCR was conducted in triplicate with 2 μl of isolated DNA using the SsoAdvanced Universal SYBR Green Supermix (Biorad) and CFX96^^TM^ Real-Time System (Bio-Rad) according to manufacturer’s instruction. As primers oligonucleotids 5′-CCGAGTGCTTGCACTCAATTGG-3′ and 5′-CTCTTATGCCATGCGGCATAAAC-3′ targeting the 16S rRNA of *E. faecalis* were used in a concentration of 20 mM (Sedgley et al., 2005b). 10-fold serial dilutions of *E. faecalis* genomic DNA in a range between 10 fg and 10 ng were run in parallel for calculation of a standard curve. The standard curve was used to calculate the amount of *E. faecalis* specific genomic DNA in the tooth samples. To calculate chromosomal copies numbers of *E. faecalis* DNA, this amount of genomic DNA is divided by the weight of a single molecule of chromosomal DNA of *E. faecalis*. The weight of a chromosomal DNA molecule is calculated as m = *n*[1 mol/6 × 10^23^ (bp) [660 (g)/mol] = *n*(1.096 × 10^–21^ g/bp)], where m is the average mass of a single genomic DNA molecule and n is the genome size. With a genome size of 2,939,973 bp for *E. faecalis* this leads to a genomic mass of 3.22 fg.

For determination of colony-forming units (CFU), 10 mg of tooth powder was dissolved in 100 μl of 0.9% NaCl. Serial dilutions were then plated on TSB agar plates.

### Lysozyme and Phosphate Assays

To determine lysozyme activity, 20 mg/ml lysozyme was dissolved in Tris-HCl buffer containing indicated concentrations of EDTA and incubated at 37°C for indicated time spans. Lysozyme activity was then measured with the EnzChek Lysozyme assay kit (Invitrogen) according to manufacturer’s instructions (Hannig et al., 2005, 2013). Samples were diluted appropriately in the provided reaction buffer to be within the linear range of the assay.

To determine phosphate concentrations, 10 mg tooth powder was incubated with 200 μl of a solution containing the indicated concentration of EDTA for the indicated time at 37°C under agitation. Afterward, tooth powder was pelleted and supernatants were used for the phosphate assay (Hertel et al., 2017; Kensche et al., 2019). Here, 10 μl of sample were mixed with 200 μl malachite green. The reaction was incubated for 15 min and then absorbance at an optical density of 650 nm was measured with a Tecan infinite M200 microplate reader. Serial dilutions of 3.075 mM Na_2_HPO_4_ were used to generate a standard curve. Samples were diluted appropriately so that concentrations were within the linear range of the assay. Experiments were repeated in triplicate.

### Statistics

Statistical analyzes were performed with Excel and SPSS.

## Results

Several studies used molecular biological methods for quantification and evaluation of microbial colonization in dental and other hard tissues (Özok et al., 2012; Antunes et al., 2015; Siqueira et al., 2016; Qian et al., 2019). However, it is challenging to isolate DNA of high quality and quantity from these materials and often methods are not detailed and evaluated. Hence, we present here a versatile and stringent method for isolation of microbial DNA from dental materials.

### Biofilm Model for Infected Root Canals

**FIGURE 1:**
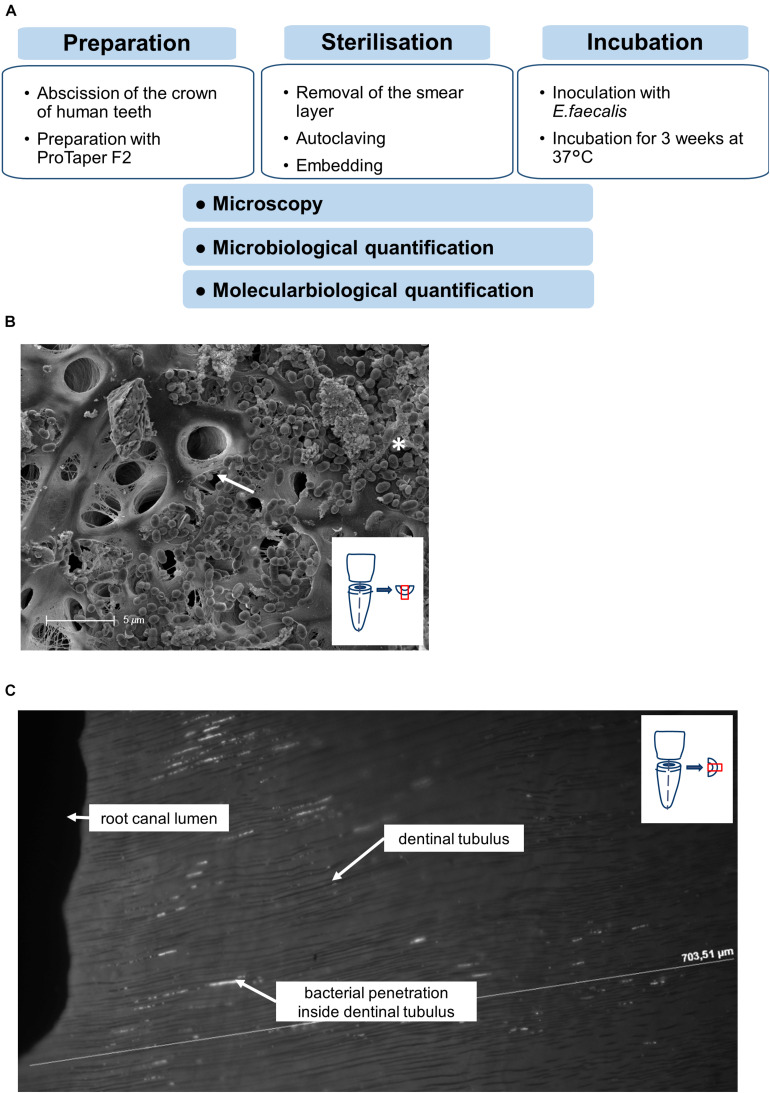
Workflow of experimental setup. **(A)** Workflow of the preparation, sterilization and incubation of teeth. **(B)** Representative SEM-image showing the colonization of the root canal surface as well as the dentinal tubules (arrow) covered with *E. faecalis* cells and biofilm formation (asterisk). **(C)** Longitudinal section of the root dentin along the dentinal tubules from the root canal lumen to the root surface, DAPI-Staining visualizing the bacterial penetration depths along the dentinal tubules.

As a model system, root canals of human premolars were colonized with *E. faecalis*, a pathogen commonly found in infected root canals. To standardize the procedure, the crowns of the teeth were separated from the roots. Then the roots were prepared with Pro Taper Gold F2 (Dentsply) under irrigation with sodium chloride (0.9%) and EDTA (20%) for 1 min to remove the smear layer (Figure 1A). After preparation, the roots were purified from the irrigants by an ultrasonic bath in aqua dest. for 1 h. Afterward, the teeth were sterilized by autoclaving and embedded in agarose. Then the root canals of the teeth were inoculated twice on consecutive days with 1.5 × 10^8^ CFU of an overnight culture of *E. faecalis* with a 30-gauge needle The teeth were incubated for 3 weeks under daily change of TSB culture medium under aerobic conditions to allow *E. faecalis* to form fully developed biofilm structures and to infiltrate dentinal tubules.

Successful colonization as well as formation of biofilm-like structures and infiltration of *E. faecalis* into dentinal tubuli was verified by microscopical methods. Scanning electron microscopy showed dense colonization of root canals with developing biofilm-like structures as well as bacteria invading into dentinal tubules (Figure 1B). To verify invasion of the bacteria into the tubules, also cross sections of invaded root canals were visualized by DAPI staining (Figure 1C). We could detect bacteria invading into tubules up to at least 200 μm. This proves that our model allows solid bacterial colonization of root canals and dentinal tubuli resembling naturally infected root canals.

### Disruption of Samples

**FIGURE 2:**
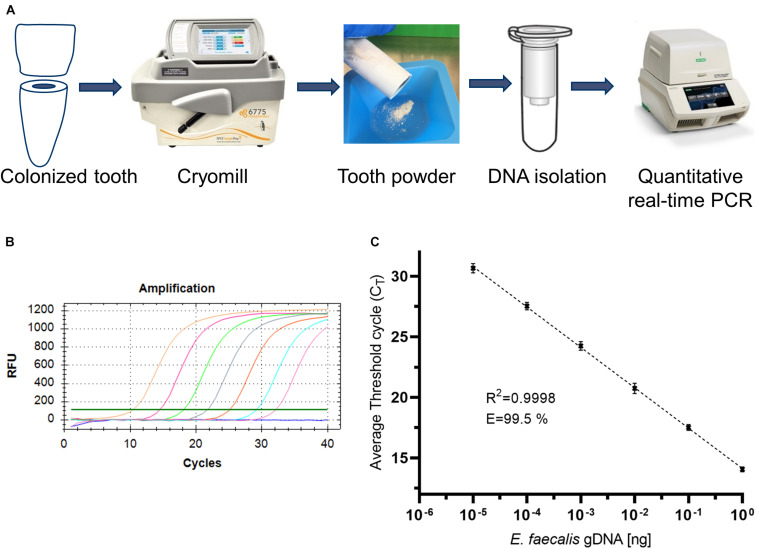
Workflow for the isolation and quantification of bacterial DNA and validation of quantitative PCR. **(A)** Workflow for the isolation and quantification of bacterial DNA. **(B)** Representative amplification plot of standard genomic DNA of *E. faecalis*. **(C)** Representative standard curve of qPCR amplification.

To disrupt samples for isolation of DNA, teeth are best grinded by cryogenic milling (Figure 2A). Thereby the dental material is disrupted into a fine powder under constant cooling at −196°C. For efficient purification of samples, it is essential to disrupt the material into a very fine powder. This improves release of bacteria that infiltrated dentinal tubuli. Furthermore, tooth powder may clog filters used for silica-based purification columns. Hence, it is recommended to use multiple grinding cycles. According to manufacturers’ instructions as well as pilot experiments, we use four cycles under the following conditions: Precool 10 min, Run Time 1 min, Cool Time 1 min, Impactor Rate 12.

### Quantitative PCR

The tooth powder was then used for isolation of DNA (Figure 2A). However, accessibility of bacteria for lysis and subsequent DNA-isolation via silica-based purification columns is limited, hence leading to low yields. Therefore, several optimization steps were included as described in the following. Isolated DNA was quantified via quantitative PCR. Oligonucleotids were specific for amplification of a fragment of 16S rRNA of *E. faecalis* (Sedgley et al., 2005a). We first tested the employed oligonucleotides by amplification of varying amounts of isolated genomic DNA of *E. faecalis* (Figure 2B). When plotting log-values of standard concentrations to C_*q*_-values, we could detect a linear correlation with an *R*^2^-value > 0.99 (Figure 2C). PCR-efficiency reached more than 99%. This shows that these oligonucleotides work well. Therefore, subsequent analyzes were conducted with this pair of oligonucleotides for qPCR.

### Decalcification Procedures

**FIGURE 3:**
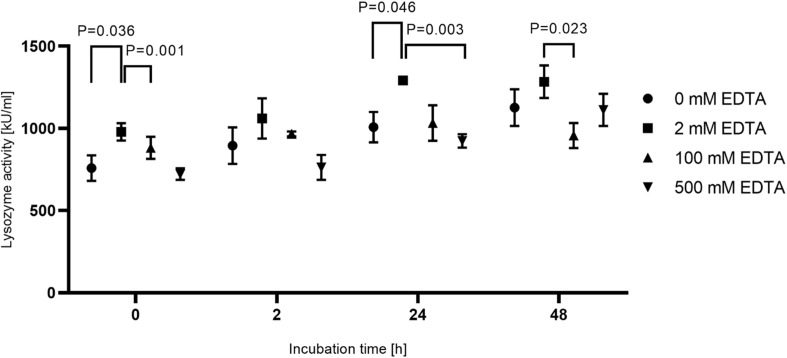
Activity of lysozyme under different incubation conditions. 15 mg tooth powder was incubated for 0, 2, 24, and 48 h at 37°C with 20 mg/ml lysozyme in 20 mM Tris-HCl pH 8.0 and 1.2% Triton-X 100 with EDTA concentrations of 0, 2, 100, and 500 mM. Shown is the lysozyme activity in kU/ml. *P* < 0.05 compared to the activity in 2 mM EDTA was considered significant. The experiment was repeated in triplicate.

**FIGURE 4:**
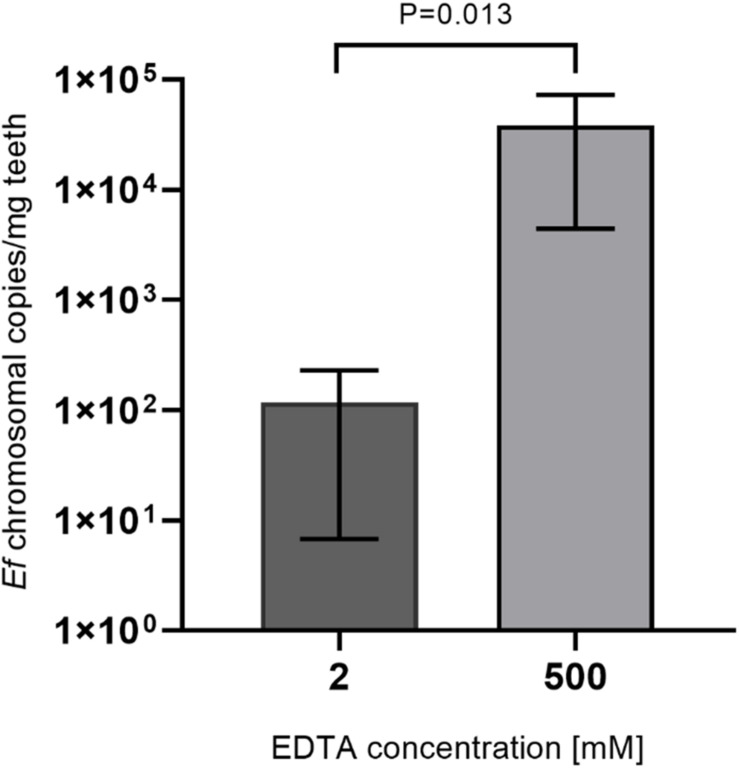
Comparison of lysozyme solutions with different EDTA concentrations. 15 mg tooth powder was incubated with lysozyme solution with 2 or 500 mM EDTA for 24 h. Then DNA was extracted and the number of bacterial chromosomes/mg tooth powder was determined by qPCR. *P* < 0.05 was considered significant.

To increase accessibility of bacteria to DNA purification, dental hard tissue is decalcified by strong chelators like EDTA. To ensure activity of lysozyme in the presence of high EDTA concentrations, lysozyme activity was measured in the presence of 2, 100 and 500 mM EDTA or in the absence of EDTA for up to 48 h incubation at 37°C (Figure 3). While activity of lysozyme was highest for all incubation periods with 2 mM EDTA, only a minor reduction of activity was detected in the presence of 100 or 500 mM or in the absence of EDTA. In addition, lysozyme activity did not decline over time suggesting a high stability of the enzyme. Overall, these results suggest that enzyme activity is not significantly influenced by EDTA and by prolonged incubation periods at 37°C. However, applying 500 mM EDTA compared to 2 mM EDTA during lysozyme treatment for 24 h increased the yield of isolated *E. faecalis* specific DNA by more than 200-fold as assessed by qPCR (Figure 4). This indicates that decalcification by high concentrations of EDTA leads to a major increase in DNA recovery rates.

**FIGURE 5:**
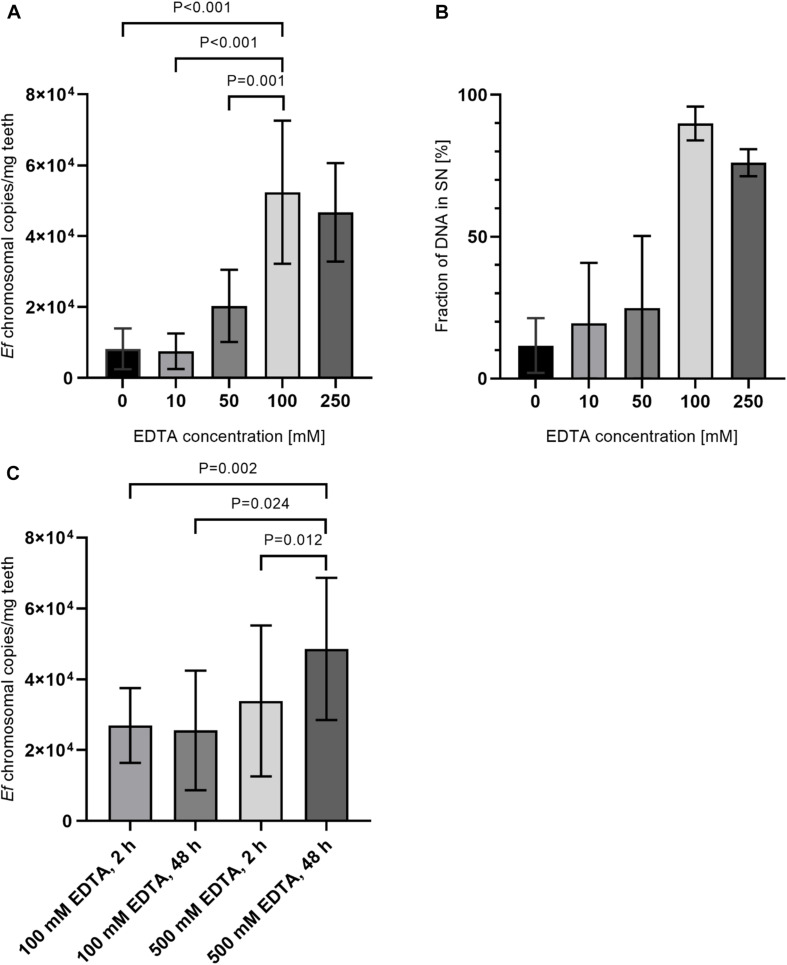
Comparison of preincubation with different EDTA concentrations and incubation times. **(A)** 15 mg tooth powder was incubated with five different EDTA concentrations for 24 h. Afterward, samples were split into a pellet and supernatant fraction as described in the manuscript. Samples were then treated with lysozym solution containing 500 mM EDTA for 2 h. **(B)** Shown is the relative amount of DNA extracted from supernatant fraction compared to the pellet fraction. **(C)** 15 mg tooth powder was incubated with 100 mM EDTA for 2 h and lysozyme solution with 100 mM EDTA for 2 h; 100 mM EDTA for 48 h and lysozyme solution with 100 mM EDTA for 72 h; 500 mM EDTA for 2 h, and lysozyme solution with 500 mM EDTA for 2 h or 500 mM EDTA for 48 h and lysozyme solution with 500 mM EDTA for 72 h. Then DNA was extracted and the number of bacterial chromosomes/mg tooth powder was determined by qPCR. *P* < 0.05 was considered significant.

Since an increase in DNA recovery rates with incubation in 500 mM EDTA was detected, an additional preincubation step with EDTA before lysozyme treatment was added. Therefore, tooth powder was decalcified with varying concentrations of EDTA for 24 h before a lysozyme-treatment with an EDTA-concentration of 500 mM EDTA for 2 h. In this step, samples were split into a fraction containing the tooth powder and a supernantant fraction right after decalcification. These fractions were then used independently for DNA purification and the overall yield was calculated as a sum of both fractions. Here we could detect a steady increase in yield of *E. faecalis* specific isolated DNA up to 100 mM EDTA (Figure 5A). When plotting the fraction of DNA isolated from the supernatant fraction to total recovered DNA, a higher percentage of DNA is isolated from the supernatant fraction with increasing EDTA concentrations (Figure 5B). Hence, improved recovery rates are probably due to bacteria switching to the supernatant fraction after decalcification of hydroxyapatite. This may lead to better accessibility of bacteria for lysis reagents. Additionally remaining tooth powder might inhibit DNA-purification procedures. Decalcification efficiency and accessibility of bacterial DNA should increase with incubation times for decalcification steps. Also higher concentrations of EDTA should be beneficial to avoid saturation of EDTA-Ca^+^ complexes. Hence, decalcification steps were carried out for 48 h with 500 mM EDTA and afterward both the pellet and supernatant fraction were treated with lysozyme solution containing 500 mM EDTA for 72 h. As a control, the experiment was also conducted with aliquots of the same samples with 100 mM EDTA as well as shortened incubation times of 2 h again with 100 and 500 mM EDTA. Here extraction efficiency was increased by approximately twofold with longer incubation times and higher EDTA concentrations compared to shorter incubation times and/or lower EDTA concentrations (Figure 5C).

**FIGURE 6:**
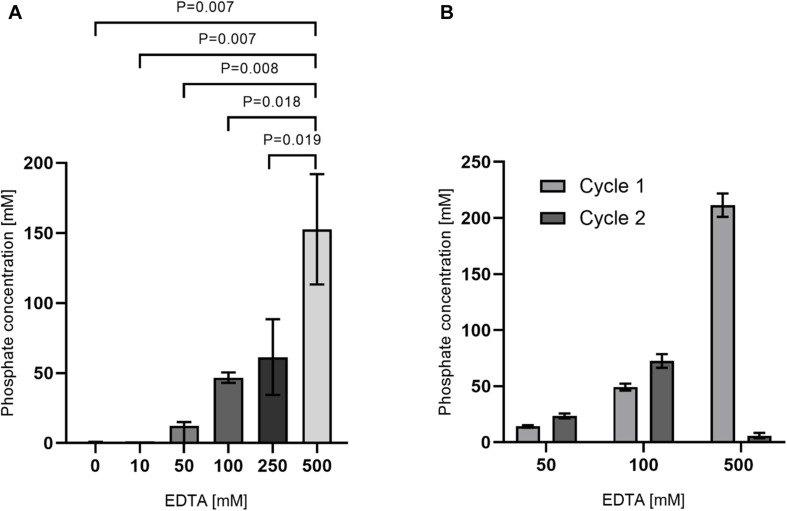
Release of phosphate after decalcification with EDTA. **(A)** 10 mg tooth powder was incubated with the indicated EDTA-concentration for 48 h. **(B)** 10 mg tooth powder was incubated with the indicated EDTA-concentration for 24 h (cycle 1). After removal of supernatants, the tooth powder was re-incubated for an additional 24 h (cycle 2). Phosphate concentration was then measured from supernatants. *P* < 0.05 was considered significant and four teeth were analyzed.

As a measurement for decalcification rates, release of Ca^+^ ions can be determined, but in the presence of high concentrations of EDTA, measurement of calcium ions is not possible. But hydroxyapatite [Ca_5_(PO_4_)_3_(OH)] is composed of calcium and phosphate ions in a ratio of 5:3. Hence, we instead measured the concentration of phosphate after decalcification in supernatants. Therefore, tooth powder was incubated for 48 h with varying concentrations of EDTA. Concentrations of phosphate were then measured with a well-established assay using malachite green. Here we could detect an increase of liberated phosphate ions with increasing EDTA concentrations (Figure 6A). In a second set of experiments, tooth powder was decalcified with 50, 100, or 500 mM EDTA for 24 h. Here we could again see an increase in phosphate release with increasing EDTA concentrations. The tooth powder was then again decalcified with the same concentrations of EDTA for 24 h. Here similar amounts of phosphate was released with 50 or 100 mM EDTA compared to the first cycle (Figure 6B). However, with 500 mM EDTA almost no phosphate was released in the second cycle. These results indicate that 500 mM EDTA lead to an almost complete dissolving of hydroxyapatite while with lower concentrations saturation is reached when all EDTA molecules formed a complex with calcium ions. Assuming an average hydroxyapatite content of 75%, 10 mg tooth powder contains approximately 15 μmol of hydroxyapatite and hence 45 μmol of phosphate. The phosphate assay has a volume of 210 μl hence leading to a concentration of approximately 213 mM phosphate. This is also in line with a complete decalcification by 500 mM EDTA where we measured phosphate concentrations in that range.

### Optimized Method for Isolation of Microbial DNA From Root Canals

**FIGURE 7:**
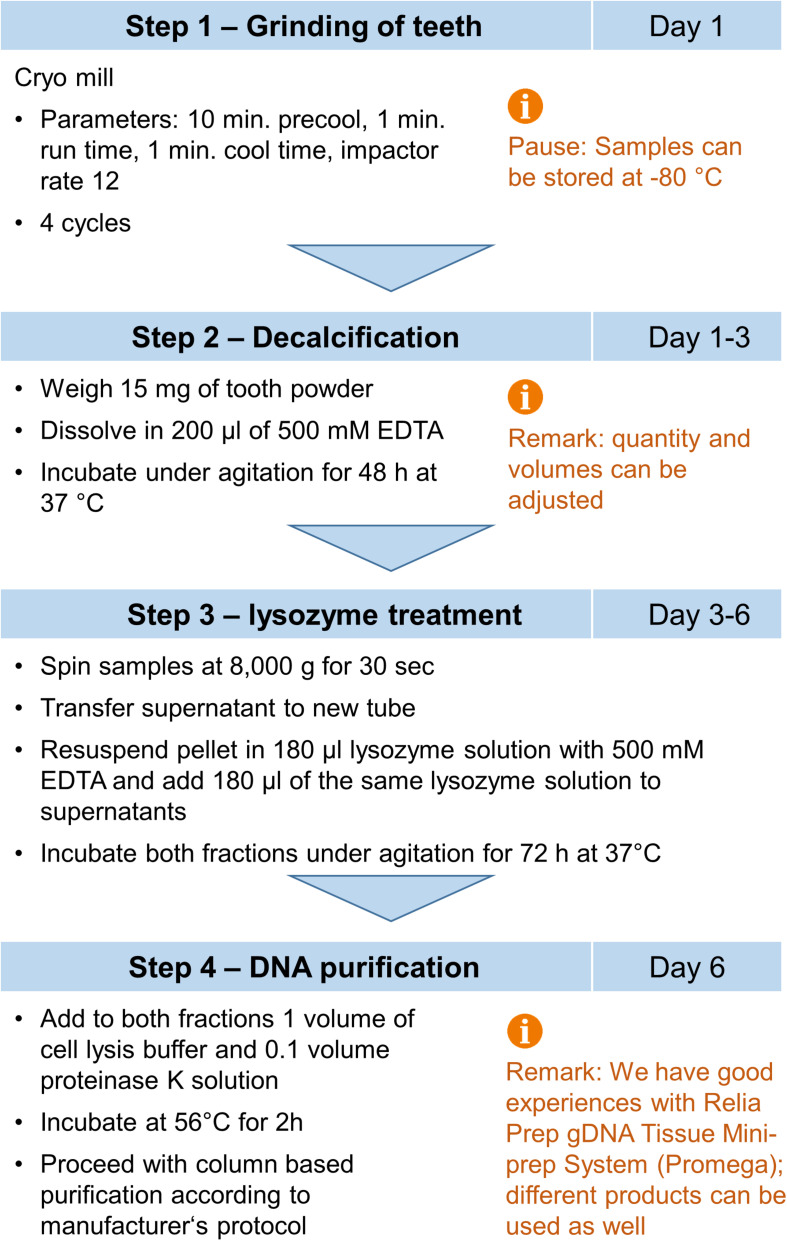
Flow chart for purification of microbial DNA from infected root canals.

In summary, we decided for the following protocol for isolation of bacterial DNA from dental hard tissue (Figure 7). First, the tooth are grinded with a cryo mill. Then a defined amount of teeth powder is resuspended in 500 mM EDTA and incubated for 48 h at 37°C under agitation. Afterward, the solution is briefly centrifuged and split in a “pellet” fraction containing the decalcified tooth powder and a supernatant fraction. The pellet fraction is resuspended with lysozyme solution containing 500 mM EDTA, while 1 volume of the same lysozyme solution is added to the supernatant fraction. Both fractions are then incubated for 72 h at 37°C under agitation. Then lysing buffer is added and DNA from both fractions is purified according to manufacturer’s instructions.

### Stability of Genomic DNA in Tooth Powder

**FIGURE 8:**
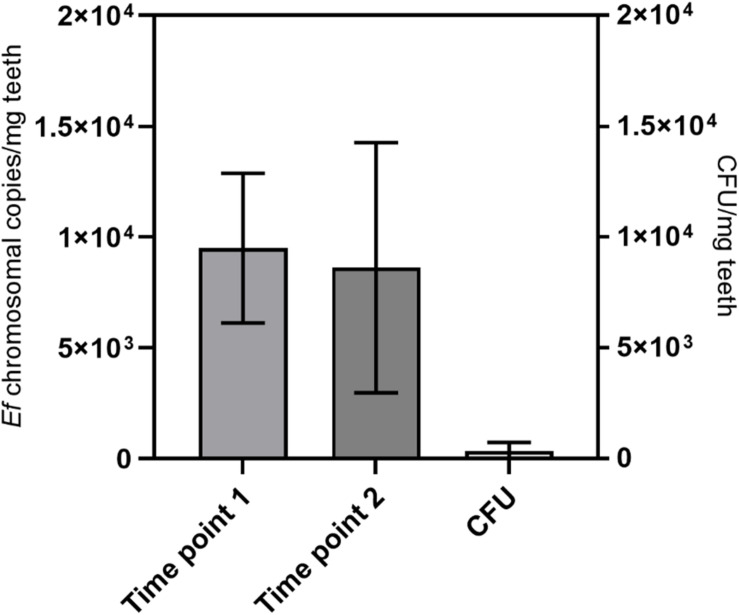
Long-term stability and comparison with CFU. DNA was extracted from teeth with root canals colonized with *E. faecalis* with the protocol from Figure 7. This was done at a first time point (time point 1) and after storage of tooth powder for 10 months at −80°C (time point 2). The number of bacterial chromosomes/mg tooth powder was determined by qPCR. On the right colony-forming units from the tooth powder was determined by platting of serial dilutions (CFU). Seven teeth were analyzed.

Next, the protocol was again tested with eight samples of experimentally infected root canals. In this case, the root canals were incubated with *E. faecalis* for 6 weeks. DNA was isolated with the aforementioned protocol and we could detect on average around 1 × 10^4^ copies of bacterial genomes/mg of tooth powder (Figure 8). Long-term storage of grinded tooth samples may be an issue. To test the stability, grinded tooth powder from these samples were stored for 10 months at −80°C. From these samples, DNA was then re-extracted from new aliquots of tooth powder and the number of bacterial chromosomes determined by qPCR. However, we could not detect statistically significant differences in copy numbers suggesting that grinded tooth powder of infected teeth can be stored for extended time periods at −80°C without impacting DNA isolation or quality of isolated DNA. Finally, copy numbers of detected *E. faecalis* genomes were compared to colony-forming units detected by plating of serial dilutions from defined amounts of tooth powder resuspended in PBS. Here CFU numbers determined by plating were approximately 25-times lower than copy numbers determined by quantitative RT-PCR. This suggests that many bacteria cannot be recovered by platting since they may be trapped within dentinal tubules or some may not survive the grinding procedure. This suggests that molecular biological methods are superior to platting in determining microbial colonization within dental hard tissues.

## Discussion

In this manuscript, we present an optimized DNA extraction procedure from dental hard tissues for molecular biological quantification of microbial colonization. Several methods for extraction of microbial or host DNA from infected teeth were published before. These include for example forensic or archeological studies (Rohland and Hofreiter, 2007; Adler et al., 2011; Raimann et al., 2012; Liu et al., 2018) or microbiome studies from infected root canals (Alves et al., 2009; Antunes et al., 2015; Siqueira et al., 2016). Many of them include disruption of teeth by cryogenic grinding. This method offers the advantage of grinding tissue to a fine powder without heat development caused by mechanical forces due to cooling with liquid nitrogen. For many study types like microbiome studies, it is not essential to purify DNA fully as long as the sample is representative. However, for quantification of DNA it is necessary to recover DNA to its full extent as much as possible.

Many studies were conducted to determine the microbiome of infected root canals either by classical culture-based methods or by modern next-generation sequencing approaches. Some of these studies collected infected material by classical methods like paper-tips (Rôças and Siqueira, 2008; Anderson et al., 2012, 2013; Tzanetakis et al., 2015; Sánchez-Sanhueza et al., 2018). Such a sampling procedure however leads to a biased collection of biofilm material readily accessible while bacteria hidden in the dentinal tubules will generally be ignored. While many studies also use cryo mills for disruption of dental hard tissue before isolating DNA, these studies generally did not use optimized DNA isolation procedures (Özok et al., 2012; Siqueira et al., 2016; Keskin et al., 2017; Qian et al., 2019). These approaches will lead to a more comprehensive DNA collection. However, still bacteria hidden in harder to approach locations like dentinal tubules will probably be under-represented, hence still causing a bias. In a similar fashion, microbiome studies of supra- or subgingival plaque often have a bias due to sampling procedures (Camelo-Castillo et al., 2015; Wolff et al., 2019). While of course whole teeth are rarely available, if they are available DNA-extraction from whole teeth may be advantageous to avoid bias due to sample collection.

Several other studies purified DNA from grinded tooth material without further decalcification or other disruption or homogenization steps. Often tooth powder was directly used for column-based DNA purification without additional preparation steps (Alves et al., 2009; Antunes et al., 2015; Siqueira et al., 2016). However, we could show that extensive decalcification of the material to disrupt hydroxyapatite is crucial for efficient DNA-purification. Some studies did use decalcification steps from grinded dental hard substances before extraction of DNA. However, these studies were generally interested in host DNA isolated from dental material, e.g., forensic or ancient DNA (Rohland and Hofreiter, 2007; Liu et al., 2018). However, these studies often deal with already partially decomposed materials but not with bacterial DNA. Still the method presented in this manuscript might also be well suited for isolation of eukaryotic DNA from dental hard tissues.

In our study, we used *E. faecalis* as a model organism since it is the most common bacterial species recovered in secondary apical periodontitis (Zargar et al., 2019). Although not shown in this manuscript, the same approach should also work for other Gram-positive or Gram-negative bacteria. Therefore, this method can be easily adapted either for other single or multispecies model systems or for clinical samples on or especially in dental hard substances. The usage of specific or universal primer pairs for quantitative PCR allows quantification of a wide range of organisms. This also allows unbiased microbiome studies via next-generation sequencing. Approaches using multispecies biofilm models and naturally infected root canals will be followed up in future studies.

A caveat of molecular biological methods is the problem that it cannot readily distinguish between vital and avital bacteria. Fluorescence-based quantification of microbiological colonization allows the staining of samples with specific dyes distinguishing between vital and avital microorganisms (e.g., staining with propidium iodide and Syto 9). Similarly, this problem is omitted by enumeration of colony forming units. However, enumeration of CFU counts from dental hard tissue is limited since scrapping of bacteria with, e.g., paper points accounts more easily accessible bacteria while bacteria entrapped in dense biofilm structures or within dentin tubuli are to large parts omitted. Similarly, disruption of material by cryogenic milling or alternative methods (e.g., with a mortar) may itself lead to reduced viability, thereby underestimating the amount of viable microorganisms. This is in concordance with our results where higher number of *E. faecalis* were enumerated by qPCR then by counting of colony forming units. It indicates that actual colonization numbers of viable bacteria are slightly overestimated by qPCR-based methods and underestimated by culture-based methods.

There are PCR-based methods available for distinguishing between DNA obtained from viable microorganisms from DNA obtained from non-viable organisms or extracelluar DNA. For example, propidium monoazide (PMA) and derivatives can be used for viability PCR (Zeng et al., 2016). These dyes can generally only enter non-viable cells while they cannot penetrate intact membranes. They bind to dsDNA and can be covalently linked to dsDNA via a photoreaction. This inhibits PCR reactions so that DNA with covalently bound PMA is not amplified in qPCR. Furthermore, potential extracellular DNA can also be excluded from analyses. Therefore, in theory only DNA originating from viable bacteria is amplified. However, this method does not always allow an absolute distinction between viable and non-viable bacteria. In some cases, cell integrity of vegetative cells may be impaired hence allowing penetration of the dye. Also increased dye concentrations and incubation times lead to penetration of small amounts of dye into bacteria with intact membranes. At the same time with low dye concentrations and short incubation times the dye will not enter non-viable cells in completion. Hence, dye concentrations as well as incubation times have to be optimized and largely depends on the species and conditions (Codony et al., 2020). However, viability PCR is a valid tool and will be integrated into the workflow in the future. In addition, RNA-based methods for discrimination between viable and non-viable microorganisms were suggested (Rôças and Siqueira, 2010). RNA-based methods are of high value for excluding non-viable bacteria from non-quantitative microbiome studies. However, due to variations in expression levels they are not suitable for quantitative analysis of microbial colonization.

It is also important to mention that non-viable bacteria are also of interest in infected root canals and dentinal tubules. Previous results demonstrated that dead bacteria can invade up to approximately 250 μm into dentinal tubules (Kirsch et al., 2017). Also the endotoxins of dead or devitalized bacteria can lead to inflammation by infiltration of macrophages (Martinho et al., 2017).

## Conclusion

In summary, we optimized the isolation of bacterial DNA from dental samples in the present study. These optimized isolation procedures are of great benefit for studies on microbial communities on and especially in dental hard tissues. Furthermore, this method can be adjusted for isolation of bacterial DNA from other infected hard tissues or materials (e.g., bones or implant materials). This is of interest both in dentistry (e.g., periimplantitis, caries), orthopedics (e.g., infected bones) and forensics (e.g., personal identification, degree of decay, and bacterial degradation). Furthermore, it also allows isolation of eukaryotic DNA from dental or bone tissue.

## Data Availability Statement

The original contributions presented in the study are included in the article/supplementary material, further inquiries can be directed to the corresponding author/s.

## Author Contributions

TS, CH, and M-TW designed the study. TS and M-TW drafted the manuscript. AP performed the experiments. TS, AP, and M-TW analyzed the data. TS, AP, MD, CH, and M-TW revised the manuscript. All authors contributed to approved the final version of the manuscript.

## Conflict of Interest

The authors declare that the research was conducted in the absence of any commercial or financial relationships that could be construed as a potential conflict of interest.
